# Stimulus Pauses and Perturbations Differentially Delay or Promote the Segregation of Auditory Objects: Psychoacoustics and Modeling

**DOI:** 10.3389/fnins.2017.00198

**Published:** 2017-04-20

**Authors:** James Rankin, Pamela J. Osborn Popp, John Rinzel

**Affiliations:** ^1^Department of Mathematics, University of ExeterExeter, UK; ^2^Center for Neural Science, New York UniversityNew York, NY, USA; ^3^Courant Institute of Mathematical SciencesNew York, NY, USA

**Keywords:** auditory perception, psychoacoustics, auditory stream segregation, dynamical systems, computational neuroscience

## Abstract

Segregating distinct sound sources is fundamental for auditory perception, as in the cocktail party problem. In a process called the build-up of stream segregation, distinct sound sources that are perceptually integrated initially can be segregated into separate streams after several seconds. Previous research concluded that abrupt changes in the incoming sounds during build-up—for example, a step change in location, loudness or timing—reset the percept to integrated. Following this reset, the multisecond build-up process begins again. Neurophysiological recordings in auditory cortex (A1) show fast (subsecond) adaptation, but unified mechanistic explanations for the bias toward integration, multisecond build-up and resets remain elusive. Combining psychoacoustics and modeling, we show that initial unadapted A1 responses bias integration, that the slowness of build-up arises naturally from competition downstream, and that recovery of adaptation can explain resets. An early bias toward integrated perceptual interpretations arising from primary cortical stages that encode low-level features and feed into competition downstream could also explain similar phenomena in vision. Further, we report a previously overlooked class of perturbations that promote segregation rather than integration. Our results challenge current understanding for perturbation effects on the emergence of sound source segregation, leading to a new hypothesis for differential processing downstream of A1. Transient perturbations can momentarily redirect A1 responses as input to downstream competition units that favor segregation.

## 1. Introduction

**Figure 1:**
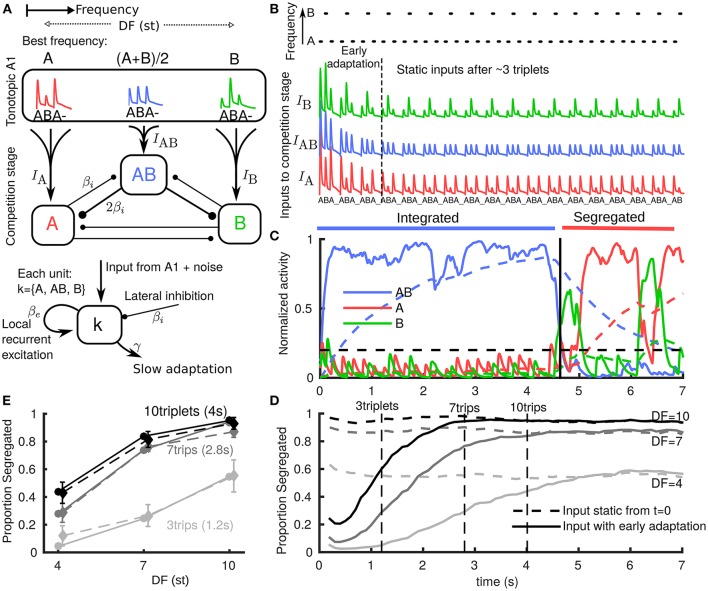
**Neuromechanistic model captures initial bias to integration and build-up of stream segregation. (A)** Model schematic with two stages: tonotopic A1 and a competition stage (downstream of and pooling inputs from A1). A1 encodes only stimulus features, while the downstream competition stage encodes percepts. Inputs from lower frequency A and higher B tones generate onset-plateau responses in A1 dependent on difference in frequency (DF) in semitones (st). In the competition stage three units encode the integrated percept (AB), the segregated A stream, and the segregated B stream. Units are in competition through mutual inhibition, pool inputs from A1, have recurrent NMDA excitation (timescale 70 ms) and undergo slow adaptation (timescale 1.4s). **(B)** (top) Stimulus paradigm where low A tones, high B tones and silences (_) each of 100 ms repeat in an ABA_ triplet pattern. (below) A1 responses to tones adapt rapidly (timescale 500 ms) with tonotopic dependence emerging and overall amplitude reducing during first 2–3 triplets. Vertical offset for visualization only. **(C)** One model simulation showing the activation threshold (horizontal dashed), and each population's NMDA variable (solid, pulsatile inputs appear smoothed in sub-threshold activity) and adaptation variable (dashed). When the central AB unit is active (integrated), the peripheral units are suppressed through mutual inhibition. Increasing adaptation for AB increases the probability of noise inducing a switch; when units A or B become active and dominant after ~ 4.5 s (segregated), the integrated (AB) unit is suppressed. **(D)** Build-up function computed as time-binned trial-averaged proportion segregated computed from *N* = 500 model simulations. With no early adaptation of inputs from A1 (input static), there is no build-up and stable proportion segregation from long-term alternations is reflected at onset. Early adaptation of inputs from A1 gives initial bias toward integrated and proportion segregated gradually builds up to DF-dependent value of long-term alternations. **(E)** Snapshots from build-up after 3, 7, and 10 triplets from model (each solid curve in E corresponds to a dashed vertical line in **(D)** are compared with psychoacoustic data (*N* = 8 normal hearing subjects) with percept reported at the end of presentation (dashed curves; errorbars show s.e.m., same for all plots).

A valued paradigm for studying auditory streaming involves segregating two interleaved sequences of A tones and B tones, distinguishable by a perceived difference in pure tone frequency and timing. The tones are organized in a repeating ABA_ABA_…pattern (van Noorden, 1975) (“_” represents silence) (Figure 1B, top). At first heard as a one stream rhythm (integrated percept), the probability of hearing two streams (segregated percept) gradually builds up over several to tens of seconds (build-up) (Anstis and Saida, 1985; Bregman, 1994; Carlyon et al., 2001). Build-up occurs more rapidly with a large difference in frequency (DF) between A and B and at faster presentation rates. However, abrupt change in the incoming sound (e.g., a step change in location, loudness or timing) can reset perception to integrated (Anstis and Saida, 1985; Rogers and Bregman, 1993, 1998), after which multisecond build-up begins again. The first perceptual switch, typically from integrated to segregated, is followed by persistent alternations between the two interpretations (Pressnitzer and Hupé, 2006; Denham et al., 2013). Build-up progresses not just to the segregation, but to the stable probability of segregation in the subsequent long-term alternations.

Neural responses to triplet stimuli have been studied in primary auditory cortex (A1) of awake monkeys (Fishman et al., 2001, 2004; Micheyl et al., 2005), in forebrain of awake (Bee and Klump, 2004; Bee et al., 2010) or behaving (Itatani and Klump, 2014) songbirds, and in the auditory periphery of anesthetised guinea pigs (Pressnitzer et al., 2008). The tonotopic organization of A1 and increased forward masking at higher presentation rates (Fishman et al., 2001, 2004) can explain the feature dependence of these responses. Studies comparing neural response data with build-up functions from human psychoacoustic experiments have shown that a trial averaged neurometric function can be tuned to match trial averaged behavioral data (Micheyl et al., 2005; Pressnitzer et al., 2008; Scholes et al., 2015). However, no study has claimed that the neural substrate for the perceptual state or switches in perceptual states lies in or before A1. Indeed, the only animal study with neural data recorded from behaving animals (Itatani and Klump, 2014) concluded that only stimulus features and not perceptual choice is encoded in songbird forebrain (analogous to A1). Responses to tones in A1 show rapid adaptation in the first few hundred milliseconds (1–3 triplets) (Micheyl et al., 2005). In this initial phase, response amplitude adapts and dependence on DF emerges (at first little tonotopic dependence is evident for tones separated by less than an octave). The relationship between this rapid adaptation (~ 500 ms) and the slower build-up process (several seconds) remains unexplained.

In Rankin et al. (2015) we developed a neuromechanistic model of auditory bistability based on a conceptual model proposed in Fishman et al. (2001). Far apart A and B tones drive tonotopically segregated representations, but for smaller DF the receptive fields overlap, leading to a common drive for an intermediate population encoding integration (Figure 1A). Our model mimics the periodic, pulsatile responses and stimulus feature dependence from A1 (Micheyl et al., 2005), which are pooled as inputs to a competition stage residing downstream (A1 encodes only stimulus features, not the percepts). At the competition stage peripheral units A and B encode segregation and a central unit AB encodes integrated. The competition network incorporates the mechanisms of mutual inhibition, slow adaptation and additive noise shown to play an important role in perceptual bistability (Moreno-Bote et al., 2007; Shpiro et al., 2009). Recurrent excitation with an NMDA-like timescale links responses and thereby percepts across silent gaps between tones and triplets (Figure 1C). Our model captures the complex dynamics of perceptual alternations, reproducing characteristic features such as the log-normal distribution of perceptual durations as well as dependence of perceptual durations on parameters such as DF (Rankin et al., 2015). We focused previously on the alternations after the first perceptual switch; the initial bias to integrated and build-up were not addressed.

Here, we propose that the initial integration bias is determined by early broad tonotopic tuning of neuronal responses in A1, while the multisecond timescale of build-up is due to slow adaptation downstream of A1. Recovery of early adaptation, say after a stimulus pause, can further explain the reset to the integrated percept. Furthermore, we find in new experiments a class of transient perturbations (single unexpected tones in the ongoing stimulus) that subsequently promote segregation, in contrast to the widely reported resets to integrated. Our model, motivated from neurophysiological studies, provides a mechanistic explanation for build-up and resetting whilst also accounting for new experimental findings.

## 2. Results

### 2.1. Neuromechanistic model explains initial bias to integration and build-up of stream segregation

In order to study build-up in our existing model, we made one change to the inputs based on further observations about the early responses to triplets in A1 (Micheyl et al., 2005). We introduced rapid adaptation (timescale 500 ms) for both input amplitude and DF dependence (Figure 1B). During the first 2–3 triplets input evolves as if driven by a DF that is effectively small but gradually increasing to a static value. The AB unit receives enough input bias to become active, suppress the peripheral units and become dominant first (Figure 1C). Time-binned build-up functions (three DF and two input cases) were computed by averaging across simulations. In the input static case (Figure 1D dashed) the inputs are assumed post fast-adaptation (Figure 1B after 3 triplets) and the time-course only reflects the static probability of post build-up alternations. In the input adapting case (Figure 1D solid) responses are initially biased to integrated and gradually build-up to the static probability of later alternations. The slower timescale of this build-up arises from the mechanisms already established in (Rankin et al., 2015) for the competition stage downstream of A1. In particular, there is a slower adaptation process at the competition stage. In psychoacoustic experiments, the build-up process can be sampled with short stimulus presentations of different lengths with percepts reported at the end. Vertical lines in Figure 1D show three such snapshots from the model (Figure 1E solid). These are compared with psychoacoustic data (Figure 1E dashed) for three DF and two presentation length conditions. A repeated measures ANOVA showed a significant effect of DF [*F*_(2, 14)_ = 37.49, *P* < 0.001], of presentation length [*F*_(2, 14)_ = 19.49, *P* < 0.005] and their interaction [*F*_(4, 28)_ = 4.34, *P* < 0.05], see Appendix A in Supplementary Material. The close match with these data show that the model accurately captures build-up (increasing segregation with both DF and presentation length). Our model is the first to produce the bias to integrated in a manner directly motivated from neurophysiology data (Fishman et al., 2001; Micheyl et al., 2005) (fast adaptation in A1) and to produce gradual build-up due to a slower adaptation timescale downstream of A1 (at the competition stage in our model).

### 2.2. Promotion of segregation by distractor and deviant tones

**Figure 2:**
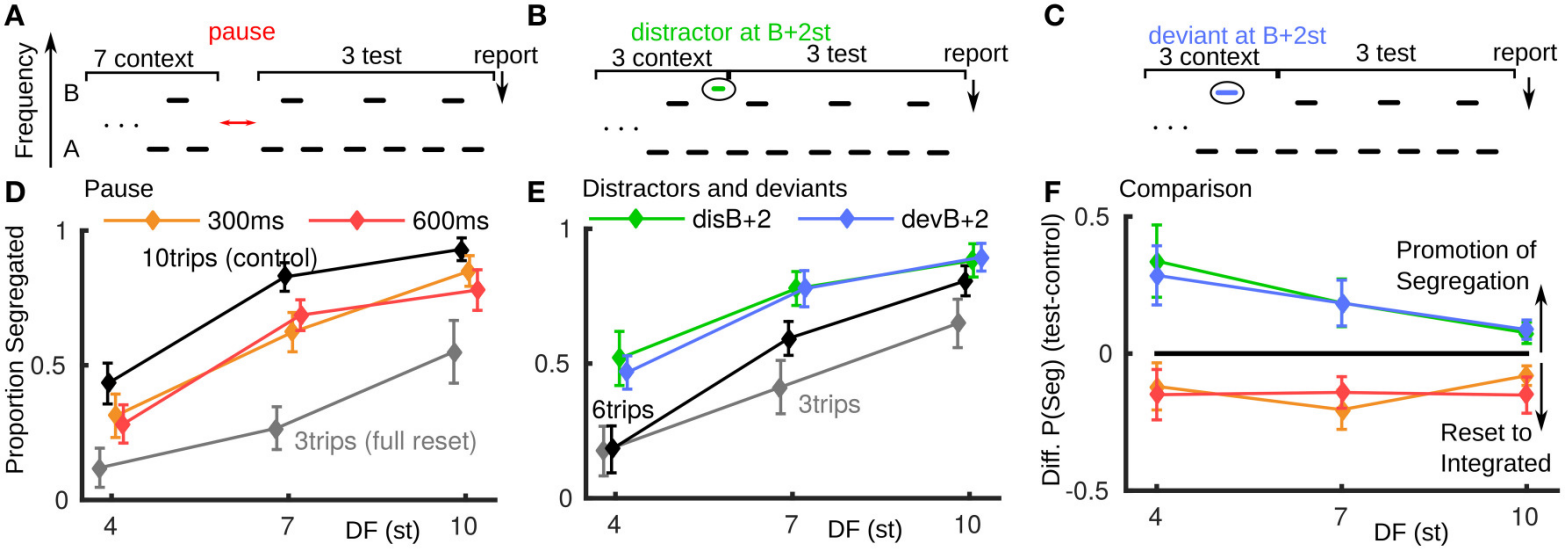
**Promotion of segregation by distractor and deviant tones, behavioral data (normal hearing subjects, ***N*** = 8)**. **(A)** Paradigm for pause of 300 or 600 ms after 7 context triplets, followed by 3 test triplets, where subjects report percept of final triplet. **(B)** Paradigm for distractor falling in the normal gap between last of 3 context triplets and first of 3 test triplets. **(C)** Paradigm for deviant B tone in last of 3 context triplets. **(D)** Brief pauses in stimulus presentation result in a partial reset to integrated. The test conditions (red, orange) would align with the 10-triplet control (black) if the pause had no effect and align with the three-triplet control (gray) for a full reset to integrated. **(E)** Both a distractor tone in the gap between triplets or a deviant tone within a triplet can promote segregation. Proportion segregation increased for all test conditions (green, blue) relative to the control condition black. **(F)** Direct comparison between stimulus pauses and distractor or deviant tones shows an opposite effect. The difference in proportion segregated between test and control conditions in **(A)** and **(B)** is plotted; when the difference is positive there is promotion of segregation, when negative a reset to integrated.

In psychoacoustic experiments we reproduced a previously reported reset toward integration for a brief pause between triplets (paradigm, Figure 2A; data Figure 2D). In all experiments described here, the stimulus ends in three normal triplets with the last triplet reported as integrated or segregated (Carlyon et al., 2003; Haywood and Roberts, 2010). In Figure 2D, if the test conditions (300 or 600 ms pause) showed no effect, the orange and red curves would align with the black ten triplet control. For a full reset to integrated the test conditions would align with the three triplet gray curve. Our results show, consistent with existing studies (Beauvois and Meddis, 1997; Denham et al., 2010), that brief stimulus pauses can result in a partial reset back toward integrated. The pause conditions had a significant effect on proportion segregated [*F*_(2, 14)_ = 5.126, *P* < 0.05], see Appendix A in Supplementary Material. The reset is of a similar magnitude for all pause duration and DF conditions.

In a new experiment six triplet presentations are used with a perturbation in the third triplet (full details in Section 5 and Appendix A in Supplementary Material). In the *distractor case* (Figure 2B), an additional tone is inserted in the normal gap between the third and fourth triplet: …ABA_ABAdABA_ …, where “d” is 2 semitones (st) higher than B. In the *deviant* case (Figure 2C), the B-tone in the third triplet is a deviant: …ABA_ADA_ABA_ …, where “D” is 2 st higher than B. A shorter presentation length was used relative to the pause experiment to avoid ceiling effects (saturation at proportion segregated = 1). See Figure 2E, where again, for no effect the test conditions would align with the black control case and for a reset to integrated, move down toward the gray three-triplet case. We found an opposite effect from pauses for a deviant or distractor tone during the ongoing triplet sequence: promotion of segregation. The increase in proportion segregated is significant for these test conditions [*F*_(3, 21)_ = 5.80, *P* < 0.05]. There is a similar effect for the deviant and distractor cases (largest for small DF). A distractor at 15 st above B showed no effect (not shown); see Appendix A in Supplementary Material.

For each experiment, by calculating the difference in proportion segregated between the test cases (colored curves) and control cases (black curves) in Figures 2D,E, we can make a direct comparison between the two types of perturbation (Figure 2F). A negative (positive) difference indicates a reset toward integrated (promotion of segregation). The promotion of segregation by a single-tone perturbation during triplets is a new and unexpected finding, opposite to the effect of pauses and other perturbations previously reported. To better understand this phenomenon, we focused on the distractor tones and further investigated their relative frequency to the triplet tones (**Figure 4F**). Before reporting these data we explore perturbations with the model.

### 2.3. Rapid recovery of adapted A1 responses explains reset to integration for pauses

**Figure 3:**
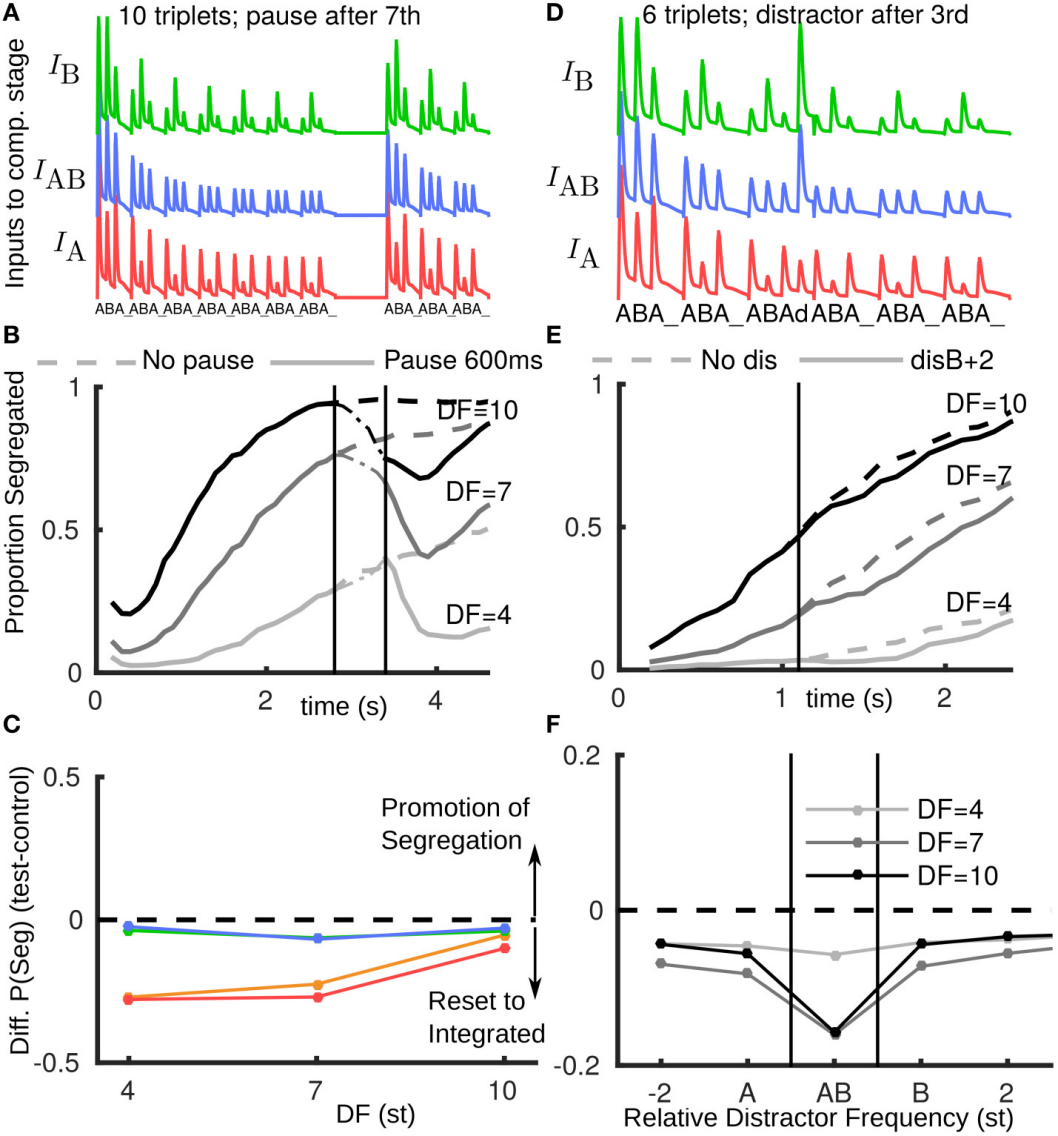
**Rapid recovery of adapted A1 neural responses explains reset to integration after pauses in the model. (A)** A1 responses fed as inputs to competition stage with a pause in presentation; after pause inputs are un-adapted. **(B)** Build-up functions from model with stimulus as shown in A (solid). Dashed curves without pause same as Figure 1D (solid). **(C)** The model captures the effect of stimulus pauses but not distractor or deviant tones (compare with Figure 2C, same color conventions). **(D)** Inputs to competition stage, with distractor d after third triplet at B+2 (2st above a normal B). Distractor tone response is assumed to have a normal tonotopic representation in A1, but be relatively more adapted at the A-location due to higher repetition rate and immediately following an A-tone offset. Distractor tone response in A1 is boosted as input to the competition stage, so the response to d is larger than for preceding tones. **(E)** Build-up function with distractor tone (solid) shows slight reset to integrated in comparison with no distractor case (dashed, as Figure 1D solid). **(F)** Across a range of tonotopic locations for the distractor tone, the model would predict a modest reset to integrated. Effect is largest when the distractor is at (A+B)/2 (labeled AB) and the DF is large, as the AB unit in the model would receive more input than peripheral units. Note x-axis does not have fixed spacing and distance between A and B changes with DF.

In the model we assume that when the stimulus resumes after even a brief pause, it will be partially recovered from adaptation (to a state similar to stimulus onset) (Figure 3A). Figure 3B shows a simulation-averaged build-up function comparing a case without a stimulus pause (input Figure 1B) to a case with a pause input (input Figure 3A). When the stimulus turns off the proportion segregated decreases (increases for DF = 4) toward 0.5. When the stimulus resumes the amplitude and effective DF of inputs from A1 have partially recovered; the proportion segregated continues to decrease (starts decreasing for DF = 4) before resuming gradual build-up. In this way, the model accounts for the partial reset toward integration across all DF conditions, compare red/orange curves in Figure 3E(model) with Figure 2F (experiments).

### 2.4. Model hypothesis on differential processing of non-triplet tones

For a distractor tone in the model, in order to compute an input amplitude, we first assumed the same rules as for the standard A and B tones. One modification was to assume a reduced response in A1 at the A-location due to higher repetition rate and the distractor immediately following an A-tone offset (stimulus-specific adaptation Ulanovsky et al., 2004; Taaseh et al., 2011). Until now the responses in A1 were taken directly as inputs to the competition stage, without modification. However, in initial simulations we found almost no effect of introducing a single new tone. A further assumption is that a distractor tone, arriving in a window where silence was expected, would be detected as a new event, and boosted (approximately to the level of an un-adapted tone) as input to the competition stage. Figure 3D shows inputs for a distractor 2 st above a normal B (B+2), see Appendix B in Supplementary Material. Still, only a small reset toward integrated is observed (Figure 3E). Using the same assumptions for a deviant B tone at B+2 we find a similar effect (Figure 3C). A comparison with the experimental data from Figure 2F shows that the model has not captured the effects of deviants and distractors. A further exploration varying relative frequency of the distractor tone (Figure 3F) shows that the model would predict a large reset toward integrated when it is at a frequency (A + B)/2, in which case the AB unit receives the most additional input from the distractor tone. However, this prediction was not borne out in later experiments.

**Figure 4:**
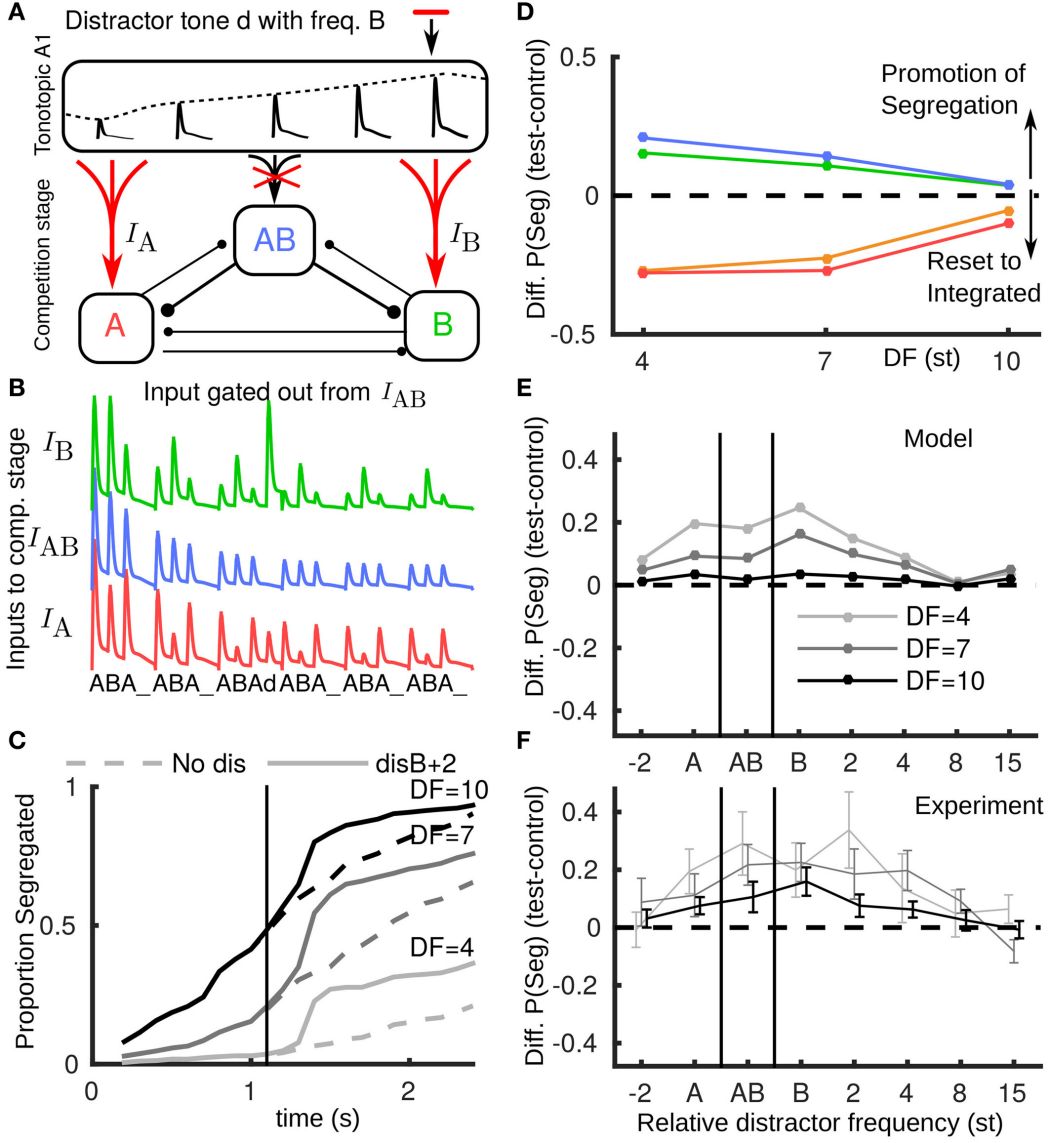
**Non-triplet (deviant or distractor) tones are gated out from AB unit. (A)** Schematic showing how a distractor d with, e.g., the frequency of a B tone, propagates in the model when boosted to A and B units and gated out from the AB unit. **(B)** Model inputs from A1 with a distractor tone (at B+2) after the third triplet where it is not seen by the AB unit, contrast with Figure 3D. **(C)** Build-up function in this case shows that the distractor tone results in an immediate increase in segregation, contrast with Figure 3E. **(D)** Based on the new assumption the model captures, along with the resetting effects of pauses, the promotion of segregation for distractor and deviant tones, compare with Figure 2C (same color conventions). **(E)** The model predicts the largest effect for the distractor tone when it is close to the B location, that the effect is largest for small DF and that the effect diminishes if the distractor tone is too far above B or below A. Note x-axis does not have fixed spacing and distance between A and B changes with DF. **(F)** Experimental data showing promotion of segregation with respect to the tonotopic location of the distractor tone.

Using the model, we tested a new hypothesis for how novel inputs, tones that are saliently not part of a triplet, propagate from A1 to the competition stage. These include tones not fitting the temporal pattern of a regular triplet (e.g., the distractor tone) or not matching the frequency of the tones in a regular triplet (e.g., a deviant tone); in informal listening either case is saliently different from a normal triplet. We suppose that the AB unit, encoding the integrated percept, will only receive inputs matching a normal triplet, while as before, the unexpected event results in boosted input to the competition stage (Figure 4A). For example, a distractor tone B+2 leads to a larger than expected input at B, but no input to AB (Figure 4B). The build-up function shows an increase in segregation (Figure 4C) due to the peripheral units receiving more input. In both the distractor and deviant cases segregation is promoted, recapitulating the behavior with the reported experimental data, compare Figure 4D (model) with Figure 2F (experiments). Note that the model captures the largest promotion of segregation occurring for small DF.

We further applied the model to predict the dependence of change in proportion segregated on the frequency of a distractor tone (Figure 4E). Predictions: (1) the promotion of segregation occurs for a range of relative frequencies for the distractor tone, (2) the effect is strongest when the distractor tone is close to the A and B tones, (3) there is no effect if the distractor is too far in tonotopy from the A and B tones, and (4) asymmetry, e.g., that the effect is more prominent when the distractor is near or above the B tone than when it is near or below the A tone. Further experiments confirmed these general trends for distractor tones at more frequencies (total 8) relative to the A and B tones (Figure 4F). One experiment tested distractors aligned with the A (disA), the B (disB) or directly between (disAB). These conditions showed a significant effect on proportion segregated [*F*_(3, 21)_ = 5.00, *P* < 0.05]. Another experiment tested distractors above B (disB+4, disB+8) and below A (disA-2). These conditions did not show a significant effect [*F*_(3, 21)_ = 2.145, *P* = 0.125], which is indicative of the diminishing effect of the distractors away from the A and B tones.

## 3. Discussion

We report new insights on the dynamics of build-up in perceptual segregation, including the initial bias toward integration, and the effects of pauses, distractor tones and deviant tones. In audition the initial percept is typically integration with segregation developing over seconds (Anstis and Saida, 1985; Bregman, 1994). But such biasing toward integration has eluded neuronally-based explanation. We suggest that the initial bias is determined by broad onset activation in neurons selective to low-level features (e.g., tone frequency Micheyl et al., 2005; Pressnitzer et al., 2008) in or even below primary sensory cortex, prior to early adaptation and emergence of strong feature dependence. This property at onset biases the initial conditions that are propagated downstream of A1 to areas involved in identification of perceptual patterns and competition between them where build-up develops more slowly. Our study focused on auditory streaming, but the principle could generalize to motion plaid displays consisting of two gratings moving in different directions, also showing an initial bias toward integrated pattern motion (Hupé and Pressnitzer, 2012). Neural responses in visual areas representing the relevant low-level feature (motion direction) show a broader initial activation and a bias toward the vector average, i.e., integrated, direction (Recanzone and Wurtz, 1999). Our experiments and modeling demonstrate that the bias in the auditory case is partially restored during pauses that allow some recovery from early and fast adaptation (as brief as sub-sec), thereby allowing a refresh of the biased initial conditions. While various changes in stimuli can also interrupt build-up and reset the percept toward integration we have discovered a class of perturbations that promote segregation rather than integration. In auditory streaming a transient perturbation that disrupts the triplet pattern (e.g., replacing B by a deviant D or adding a distractor tone d between triplets) promotes segregation. Intuitively, these events could briefly make one of the streams more salient and cause a switch. Our model provided an opportunity to seek a more mechanistic explanation. Based on our experiments and modeling we propose that non-triplet tones are processed differently downstream from primary auditory cortex. Furthermore, our results support the notion of auditory streaming being bistable between perceptual states, where a pause or aberrant tone can flip the percept in a specific direction and the perturbation's effect is still evident several seconds later.

### 3.1. Timescale of the reset to integrated

Using short stimulus presentations, we confirmed a partial reset to integrated for pauses of 300 or 600 ms, but did not find an increasing trend between the two conditions. A reset to integrated has been shown with pauses longer than 1 s using short stimulus presentations (Cusack et al., 2004; Snyder et al., 2008, 2009) and with briefer pauses (<1 s) using long stimulus presentations (during bistable alternations) (Denham et al., 2010). Sussman et al. (2007) showed a reset for multi-second pauses, using EEG recordings and a mismatch negativity paradigm.

In our model, initial A1 responses had a large amplitude and broad tonotopic tuning; fast adaptation on a common timescale led to static responses with lower amplitude and tightened tuning. The tonotopic component is key for the initial bias toward integration. A rapid recovery of the fast adaptation following a stimulus pause led to a partial reset to integrated, consistent with our data. For a long enough pause there must be a full reset to integrated, as if hearing the stimulus for the first time. Bregman (1978) suggested biasing from previous stimuli would have recovered within 6 s and this was confirmed by later studies (Beauvois and Meddis, 1997; Snyder et al., 2008). Our results suggest that although rapid recovery of adaptation in A1 may explain the partial reset to integrated (even for very brief stimulus pauses), the multi-second timescale of longer term recovery may also be related to processes downstream of A1.

### 3.2. Link between context and perturbations

A sudden change after a sequence of context triplets causes at least partial resetting of build-up back toward integration, as shown for a change in ear of presentation (Anstis and Saida, 1985), a shift in perceived loudness and/or location (Rogers and Bregman, 1993, 1998), a switch in attention (Carlyon et al., 2001; Thompson et al., 2011) and a pause in presentation (as described above); see review Moore and Gockel (2012). Like a pause, a switch in attention could allow recovery from adaptation. Otherwise a one-time shift of the entire stimulus in location or intensity (an increase, but not decrease Rogers and Bregman, 1998) could recruit previously unstimulated and, therefore, unadapted neurons. We may view the triplets preceding the deviant and distractor tones during build-up as context. Different types of context can bias perception toward (i.e., prime for) integration or segregation (Sussman and Steinschneider, 2006; Snyder et al., 2008, 2009; Rahne and Sussman, 2009). Even for a context of a single stream of tones, say A_A_A_A_, that would alone promote segregation for subsequent test triplets ABA_ ABA_…, similar disruptions as above at the end of the context sequence lead to integration, as if the effect of the context was undone (Rogers and Bregman, 1993, 1998; Beauvois and Meddis, 1997). Also, a single deviant A' at the end of an A_A_A_…context can reduce or eliminate the expected bias toward segregation (Roberts et al., 2008; Haywood and Roberts, 2010, 2013). So while these various disruptions favor integration and may a priori lead one to a generalized expectation that a single transient distractor tone (between triplets) or a single deviant tone (within a triplet) should also promote integration, we found the opposite — promotion of segregation in the subsequent triplets. Nevertheless our results do not contradict these previous studies. Studies looking at the effects of deviant tones did so by placing these at the end of a single stream context (Roberts et al., 2008; Haywood and Roberts, 2010, 2013); in our study we placed the deviant or distractor at the end of context triplets. Thompson et al. (2011) included an experiment with a single delayed-onset deviant B tone, but did not report promotion of segregation or resetting. Still, other stimulus perturbations may promote segregation. Further experiments should consider whether single-tone deviants in features other than frequency (e.g., lateralization or loudness) can promote segregation.

### 3.3. Promotion of segregation and differential processing of non-triplet events

Our model accounts for the observed segregation-promoting effects by assuming that inputs propagate to the competition stage in a differential manner: A1 responses to a deviant or a distractor tone do not reach the “integration (AB) unit.” It encodes a non-trivial rhythm and can be viewed as more sensitive to, effectively selective against, sounds that break the triplet pattern. Our implicit assumption is that the aberrant tone is identified as a mismatch and is deflected from reaching AB. Viewed differently, an incoming sound inconsistent with the integrated percept might result in the integration unit being briefly suppressed, allowing the peripheral units to take over. The crucial aspect is that the incoming tones have a differential effect on the integrated and segregated units. The effects of distractor tones also show a dependence on tonotopy, which led us to favor an input-based explanation.

Our results allow us to rule out some other potential explanations for the effects of non-triplet perturbations. Suppose that such perturbations indiscriminately cause a switch in perception away from the current percept. One might argue that we saw switches only from integrated to segregated since we considered perturbations only during build-up, when integration is thought to be dominant. However, our data do not support the idea of switches from segregated to integrated. At DF=10, where ~ 50% of trials are already segregated after 3 triplets, we saw no evidence of a reset or switch back toward integration (Figure 2E), either in the group data, or in individual subjects (not shown). This facet of the data is consistent with the proposed notion that input propagates from A1 to the segregated units, but not the integrated unit. Another hypothesis could be that any transient, salient perturbation distinct from standard triplets promotes segregation. However, our data showed no effect for distractor tones sufficiently far in frequency from the A or B tones. Our modeling work shows that this interaction could be through input from the distractor tones still propagating to segregated units with tonotopic dependence.

Haywood and Roberts (2013) showed that hearing a single A tone before the triplets could make that stream more salient. Could there be a similar effect in our data, where the distractor tone makes one of the streams more salient, or briefly directs attention toward it? The range of conditions for which we found promotion of segregation includes several cases where the perturbation is not an A or a B tone. The distractor tone d appears in a sequence …ABA_ABAdABA_…. It could be that the d is being grouped into a new triplet (AdA or B-d-B), thus making the A or B stream more salient (or highlighting their separation) ahead of the upcoming test triplets. For a distractor or deviant tone, the proposed mechanism in our model boosts inputs to the competition stage for the segregated units whilst gating out input to the integrated unit. This selective transient modulation of input gains could be viewed as a brief top-down attentional effect. However, for an attention mechanism, the selective gain would likely act in response to the perturbation mismatch with some delay. In our current model we have idealized the transmission of input from A1 to competition stage without a delay.

### 3.4. Build-up and bistability in models

Most existing computational models of auditory streaming have focused on reproducing the dependence (van Noorden, 1975) of perceptual bias on DF and presentation rate (Almonte et al., 2005; Elhilali and Shamma, 2008; Wang and Chang, 2008), the dynamics of build-up (Beauvois and Meddis, 1991; McCabe and Denham, 1996) or both (Beauvois and Meddis, 1996). A complete theoretical framework for streaming should account for build-up as well as the later alternations, given that the probability of perceiving segregation converges to the long-term probability of bistable alternations. Some recent models focused on post-build-up alternations (auditory bistability) (Mill et al., 2013; Barniv and Nelken, 2015; Rankin et al., 2015; Steele et al., 2015). The initial bias to integration is set by specifying a priori initial conditions (Barniv and Nelken, 2015; Steele et al., 2015). In Mill et al. (2013) the bias is emergent through an early stage of an algorithmic pattern discovery. Our model that accounts for alternations, and was further developed here to describe build-up, is the first treatment to explain the initial bias for integration through a direct link to observed neurophysiological responses (Fishman et al., 2001; Micheyl et al., 2005). To the best of our knowledge, no other model has been used to investigate resetting effects, or the effects of perturbations in general.

### 3.5. Future directions for our model

Our current neuromechanistic model relies on a lumped version of a distributed network, where a few discrete units pool inputs from different tonotopic locations in A1. Although this view allows the model to account for many phenomena (stimulus parameter dependence, build-up, alternations, resetting for pauses), the notion of differential processing introduced to account for promotion of segregation approaches the limit of our modeling framework, and suggests the need for a richer description. One avenue for extension would be to consider a continuous feature space in DF, as proposed in Almonte et al. (2005), at least at the A1 stage of the model. Although the rules for the tonotopic spread of A1 responses allowed us to consider, for example, distractor tones away from the three locations A, B and (A+B)/2, a more refined description would define how A1 responds in time to any combination of tones across DF (and consider other paradigms, for example, involving frequency-banded maskers Elhilali et al., 2009b). As a further extension we could introduce an additional dimension to the feature space, e.g., selectivity to different repetition rates of the streams and the relative timing (phase) of the inputs. A first step in this direction was made in Beauvois and Meddis (1996), with the use of delay lines to introduce a temporal feature space. Beyond this, a suitable theoretical framework to study might be a coupled oscillator network sensitive to frequencies in the range of the repetition rates of the tone sequences, not just the frequency of the tones (like tonotopy in A1). fMRI studies have implicated a broader network involved in streaming, including areas associated with rhythm and timing (Kashino and Kondo, 2012). The design of such a network and the necessary mechanisms for competition could build directly on our present model. Such networks have been used in studies of rhythm perception (Large et al., 2015) and in phenomenological studies of perceptual bistability (Borisyuk et al., 2009). Such a richer description would allow one to pursue the origin of the differential processing we propose here and to investigate the effects of temporal coherence, a strong cue in auditory stream segregation (Elhilali et al., 2009a).

## 4. Conclusion

Our model with the developments presented here is the first grounded in neurophysiological detail to account for build-up and subsequent bistable alternations. We propose that the initial bias to integrated arises naturally from the rapid but delayed emergence of low-level feature dependence and that the more gradual timescale of build-up comes from competition mechanisms downstream of A1. This is the first explanation of integration bias and build-up motivated directly from neurophysical data (responses to triplet sequences in A1 Micheyl et al., 2005).

New findings presented here challenge the current understanding of how the segregation of auditory objects is affected by interruptions and perturbations. A reset of the build-up process results from an established class of perturbations that shift the entire triplet stimulus in location, loudness or timing. We illustrate that the rapid recovery of responses in A1 can explain resetting for stimulus pauses. We demonstrated a new and opposite effect, promotion of segregation, by a complementary class of perturbations that transiently alter a single triplet or introduce a new non-triplet element. Our modeling in conjunction with confirmed experimental predictions led to a new hypothesis: that new non-triplet events (deviant or distractor tones) are gated out from the neural population encoding the complex integrated rhythm.

## 5. Materials and methods

### 5.1. Neuromechanistic model

The neuronal circuits for competition and perceptual encoding are assumed to be downstream and receiving inputs from A1. The periodic inputs mimic the A1-responses to ABA_ sequences reported in Micheyl et al. (2005). Neuronal activity is described by mean firing rates and competitive interactions emerge through a combination of excitatory and inhibitory connections, slow adaptation and intrinsic noise. We provide a brief outline of the model architecture, mechanisms and inputs here; the full model equations and further details can be found in Appendix B in Supplementary Material.

The schematic in Figure 1A shows downstream units *A*, *B*, and *AB* that respectively pool inputs from regions of A1 centered at locations with best frequencies A, B and the midpoint between (A + B)/2. We associate a variable *rk* (*k* = {*A, AB, B*}) with each unit representing the mean firing rate of a population of neurons in the competition network. For each unit *rk* the intrinsic dynamics are illustrated in Figure 1A and described by a differential equation like the following,

(1) $$\begin{array}{l} \tau_{r}{\dot{r}}_{\text{AB}}=-r_{\text{AB}}\;+\;F(\beta_{e}e_{\text{AB}}-\beta_{i}(r_{\text{AB}}\;+\;r_{\text{A}}\;+\;r_{B})-\;ga_{\text{AB}}\;+\\ \;I_{\text{AB}}\;+\;\chi_{\text{AB}}). \end{array}$$

By way of an example, we describe this equation for *r*_AB_ in detail; the equations for *r*_A_ and *r*_B_ take the same general form. The cortical timescale is τ*r* = 10 ms. A sigmoidal firing rate (smooth threshold) function *F* (see Appendix B in Supplementary Material) process all inputs to the unit. Local excitation *e*_AB_ has strength β_*e*_ = 0.65 and evolves on an NMDA-like timescale τe = 70 ms. Global inhibition (assumed instantaneous and so proportional to the cortical variables *r*_*k*_) has fixed strength β_*i*_ = 0.3 independent of DF. Note β_*e*_ > β_*i*_ so there is net local excitation. Linear spike frequency adaptation (slow negative feedback) *a*_AB_ has strength *g* = 0.045 and a timescale of 1.4 s.

The input *I*_AB_ mimics A1 cortical responses to triplet tone sequences; full details are given in Appendix B in Supplementary Material. There are two components to the early adaptation of these responses, both consistent with observations from Micheyl et al. (2005) and sharing a common timescale τ_A1_ = 500 ms (Figure 1B). Firstly, the overall amplitude of responses decays. Secondly, the effective DF is initially small i.e. the DF dependence of the responses takes time to emerge. After a stimulus pause, the A1 adaptation is assumed to rapidly recover (τ_rec_ = 100 ms), such that when the stimulus resumes after an adequate pause (say 2 × τ_rec_) the model inputs resemble those after initial stimulus onset (Figure 3B). For a distractor tone (or a deviant tone) input amplitude and tonotopic spread are consistent with a partially recovered response to the tone. At the tonotopic location A, responses to a distractor are reduced, because the distractor immediately follows the offset of a normal A tone (referred to as temporal forward masking in Fishman et al., 2001). Intrinsic additive noise χ_AB_ is an independent Ornstien-Uhlenbeck process for each *r_k_*.

Numerical simulations were implemented with an Euler-Murayama scheme with a stepsize of 0.5τ_*r*_. Build-up functions were computed as time-binned averages across 500 simulations. For each time bin the fraction of trials with more activity in the AB unit than the summed activities of the A and B units was taken as the measure of proportion integrated. Computations were implemented in Matlab and batch processed using the function parfor; no special computing hardware was required. In all computations, the same set of 500 randomized initial conditions and the same 500 instantiations of the noise process (i.e., frozen noise) was used for each *r_k_*. This ensures that any differences between conditions is entirely due to changes to model parameters (e.g., reflecting different stimulus properties). For example, in Figures 3B,E, the control (No pause, No dis) curves only deviate from the test simulations (Pause 600 ms, disB+2) from the time point where the perturbation is introduced.

### 5.2. Psychoacoustic experiments

Our experimental paradigm is well suited for studying the effects of perturbations on how the subsequent triplets are perceived. In all experiments presented here (with pauses, distractors or deviants) the perturbation was followed by three normal triplets and subjects reported their perception of the final triplet, roughly 1 s after the perturbation. Three triplets provides enough stimulus duration to make a reliable perceptual judgment (Carlyon et al., 2003; Haywood and Roberts, 2010). This design precludes the possibility of subjects reporting, say, a distractor tone as being its own segregated stream, as the distractor occurs well before the final triplet. If continuous perceptual reports were used, confusion might arise about classifying an unexpected tone into its own stream at the moment the distractor is detected. A final possibility would be to use an objective measure of streaming (Roberts et al., 2008; Thompson et al., 2011). An appropriate paradigm could be the one used in Thompson et al. (2011), where performance in a deviant detection task functioned as an objective measure for streaming and showed qualitative agreement with subjective perceptual reports. In the objective task, subjects had to detect a single delayed-onset B tone and performance was best during integration. Given that the objective task relies on the detection of a delayed-onset deviant and that some trials would need to involve another deviant tone (the perturbations studied here), it could become rather confusing for a subject. It would be challenging for a subject to distinguish between multiple types of aberrant tone, ignoring some and reporting the presence of others.

#### 5.2.1. Procedure

Subjects sat in an acoustically shielded chamber and pressed keys on a keyboard to indicate their perceptual response. In each task, a short ABA_ sequence ranging between three and 10 triplets was played, and the subjects reported with button presses whether the last triplet of the sequence sounded most like the integrated percept or the segregated percept and guessed if unsure. The integrated percept was defined as hearing the A and the B tones together in a galloping rhythm, and the segregated percept was defined as hearing the A tones and B tones separately in two distinct streams. Subjects were instructed to respond as quickly as possible and had up to 5 s—the length of the inter-stimulus interval (ISI)—to respond.

#### 5.2.2. Stimuli

The repeating ABA_ triplet consist of 100 ms pure tones with 10 ms linear ramps, where the “_” indicates a silence also lasting 100 ms; in total, the duration of each ABA_ triplet is 400 ms. An inter-stimulus interval of 5 s was included between all trials. The higher frequency B tones are a variable DF semitones (st) above the lower frequency A tones. Each tone sequence was played binaurally through Etymotic headphones at 65 dB SPL. Three DF conditions were used for all experiments: DF ∈ {4, 7, 10} st. From trial to trial the A-tone base-frequencies were roved between 420 and 1060 Hz, separated by intervals of 4 st; correspondingly, the B tone frequencies ranged between 530 and 1888 Hz. The roving of base frequencies and ISI of 5 s were chosen to avoid any latent adaptation from one trial to the next (Beauvois and Meddis, 1997; Snyder et al., 2009; Sussman-Fort and Sussman, 2014).

#### 5.2.3. Subjects

Seventeen subjects in total, including one of the authors, took part in the experiments (10 female, 7 male), aged 20–51, mean age 27.9. Subjects were reimbursed for their participation and all experimental procedures complied with human subject research guidelines as approved by the University Committee on Activities Involving Human Subjects at New York University (IRB-FY2016-310). All subjects provided written informed consent and were required to pass a hearing screening.

#### 5.2.4. Conditions

The stimulus paradigm for the pause experiment is shown in Figure 2A. A total of 15 conditions (3 DF conditions crossed with 5 stimulus length/pause combinations) were tested with 20 repetitions of each condition (total of 300 trials for each of 8 subjects). Two test conditions consisted of 7 context triplets followed by a pause of 300 or 600 ms, which was then followed by 3 test triplets. Three control conditions of 3 (test only), 7 (context only), and 10 (no pause control) triplets were tested in 9 blocks of 20 trials and the test conditions in 8 blocks of 15 trials. Control and test conditions were run in separate block sections to avoid confusion about timing of perceptual reports.

Schematics of the stimulus paradigm for the distractor and deviant experiments are shown in Figures 2B,C. Three different experimental sessions, with eight subjects each, were conducted for different experimental conditions. Subjects performed 20 blocks of 15 trials each, where the length of each trial ranged from 1.2 s to 2.4 s in length. In each experiment, two control conditions included a 3 triplet and a 6 triplet condition with no deviants or distractors. Along with the two control conditions, each experiment included three distractor or deviant conditions, 6 triplets in length. Distractor tones were 50 ms in length and were inserted symmetrically in the 100ms inter-triplet gap between the third and fourth triplets of the sequence, so that there was 25ms of silence on either side of the distractor. Across the three experimental sessions, the following frequencies (in st, relative to the A and B tones of the triplets) of distractor tones were tested: A−2, A, (A+B)/2, B, B+2, B+4, B+8, B+15. Deviant tones involved a change in frequency to the B tone of the third triplet. In the one deviant tone condition tested, the B-tone was increased in frequency by 2 st.

## Author contributions

JRa and PO designed psychoacoustic experiments. PO carried out psychoacoustic experiments. JRa and PO analyzed psychoacoustic data. JRa and JRi conceived the computational model. JRa did modeling simulations. JRa and JRi wrote the manuscript.

### Conflict of interest statement

The authors declare that the research was conducted in the absence of any commercial or financial relationships that could be construed as a potential conflict of interest.
