# Supplementary material for: Stimulus Pauses and Perturbations Differentially Delay or Promote the Segregation of Auditory Objects: Psychoacoustics and Modeling
Source: Front Neurosci. 2017 Apr 20;11:198. doi: 10.3389/fnins.2017.00198 (PMC5397483; doi:10.3389/fnins.2017.00198)
Supplement: Supplementary file 1 [file DataSheet1.pdf]

Frontiers in Neuroscience

Supplemental Material: "Stimulus Pauses and Perturbations Differentially Delay or Promote the Segregation of Auditory Objects: Psychoacoustics and Modeling"†

James Rankin (james.rankin@gmail.com),
Pamela J Osborn Popp,
John Rinzel

Appendix

Figs 1–4 refer to the figures in the main paper and SIFig. 1–2 to figures here in these appendices.

A Statistical analyses for experimental results

All statistical analyses presented here utilized the software R (R Core Team 2015) with the package ez (Lawrence 2013), which produces repeated measures analysis for variance (ANOVA) while handling sphericity tests and appropriate corrections to p-values where necessary (Howell 2013).

For Experiment 1 the aim was to reproduce a known result, that brief stimulus pauses result in a partial reset towards the integrated percept. In test conditions a 300 or 600 ms pause in a ten triplet presentation was inserted after the seventh triplet, leaving three test triplets at the end. Reference control conditions (3 or 7 triplets) and the main control condition (10 triplets) reveal the behaviour with no pause (10), before the pause (7) and for the test triplets on their own (3). Data from all conditions in this experiment are shown in SIFig. 1A. A first analysis shows that the build-up is occurring for the control conditions, that is, increasing proportion segregated with DF and presentation length. An ANOVA table for repeated measures ($N = 8$, as in all experiments) within-subject factors DF and *cond* (presentation length) for the three control conditions is labelled Experiment 1A in Table 1. In this section the term *cond* represents the relevant set of conditions in each experiment, refer to the headings for each experiment in Table 1. In general, we report significant effects at the standard $\alpha = 0.05$-level and, where a Mauchly Sphericity test reached significance for the given factor, we report the Greenhouse-Geiser corrected p-value p_{GG}. The factors DF, *cond* and their interaction showed significant effects (Exp. 1A Table 1). Next, we compare the relevant control condition with the test conditions (dashed black and red/orange curves SIFig. 1A). The effect *cond* for these conditions is tested in Experiment 1B in Table 1; we found a significant effect of DF and *cond*, but not their interaction. The observed reset to integrated for short stimulus pauses is significant.

In Experiments 2–4 the effect of eight distractor cases and one deviant case were tested across three experiments. Each experiment had the same design with control conditions of 3 and 6 triplets and three test conditions (SIFig. 1B–D). In each figure the relevant comparison is between the main control condition (6 triplets) and the test conditions (color). In general the test conditions promote segregation and we report whether these effects were significant for each experiment (Table 1). For Experiment 2 there was a significant effect of DF, *cond* and their interaction. Visual inspection of SIFig. 1B suggests the significant effect of *cond* comes from increases in promotion segregated for the disB+2 and devB+2 conditions. For Experiment 3 there was a significant effect of DF, but not significant effect for *cond* or their interaction. Visual inspection of SIFig. 1B shows that the disB+4 had the largest effect. These data support the notion that distractor tones far from the As and Bs have less of an effect than those close to A and B. For Experiment 4 there was a significant effect of DF and *cond*, but not their interaction. Visual inspection of SIFig. 1B shows that the disAB condition (distractor at (A+B)/2) had the largest effect.

There was some but not complete overlap in the subjects participating in each Experiments 2–4. We therefore wanted to check for consistency in the control conditions across the experiments to ensure making comparisons for test conditions across the experiments is relevant (Fig 4F). Table 1 (bottom) shows an ANOVA table including exp (experiment number) as a between subjects factor. We found no significant effects for exp, or its interactions with other factors, confirming that comparison across these experiments is appropriate.

One might wish to apply post-hoc tests to further explore the significant effects for the variable *cond* in the ANOVAs reported above. Visual comparison of test conditions (colored curves) with the relevant control condition (dashed black curve) for each experiment represented in SIFig. 1 shows multiple cases that might be significant if tested in post-hoc comparisons. Indeed, for many of the conditions paired t-tests between the control and test conditions do reach significance at the $p < 0.05$ level. However, to rule out the chance of making Type I errors due to multiple comparisons being made (6 comparisons for Experiment 1, 9 comparisons for Experiments 2–4), it is appropriate to apply a Bonferroni adjustment to the significance levels. No conditions reach significance with the conservative Bonferroni adjustments. We note that applying a Tukey Honest Significant Differences analysis is not appropriate with our repeated measures experimental design (Howell 2013).

†Rankin J, Osborn Popp PJ and Rinzel J (2017) *Stimulus Pauses and Perturbations Differentially Delay or Promote the Segregation of Auditory Objects: Psychoacoustics and Modeling.* Front. Neurosci. 11:198. doi: 10.3389/fnins.2017.00198
http://journal.frontiersin.org/article/10.3389/fnins.2017.00198/abstract

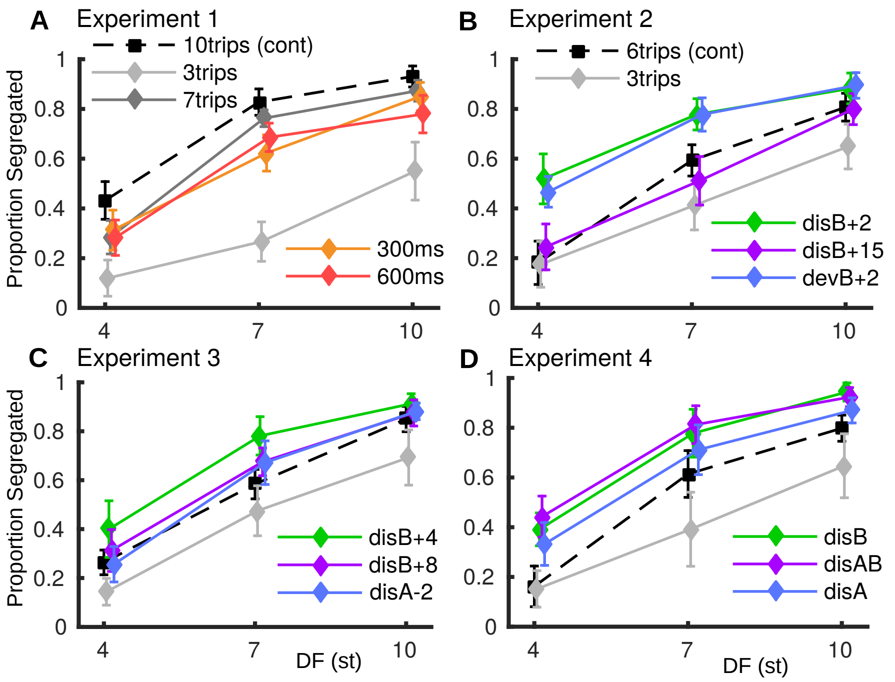

SIFig 1: **Experiments 1–4**. (A) Pause experiment with three control conditions for fixed-length presentations with indicated number of triplets (black/grey curves). Control conditions were plotted and compared with the model in Fig 1E. Test conditions with pause duration indicated (orange/red). Test conditions plotted with two control conditions in Fig 2A. (B) Distractor and deviant experiment with two control conditions with indicated number of triplets (black/grey). One deviant and two distractor cases were tested (blue/green/purple curves). One deviant and one distractor condition were plotted in Fig 2B. (C) As B for additional distractor cases tested in Experiment 3. (D) As B for additional distractor cases tested in Experiment 4. All distractor conditions from Experiments 2–4 were plotted in Fig 4F of main paper.

B Outline of the model

The network structure and neural mechanisms forming the basis of our model were originally motivated in Rankin et al (2015). In this section we give a complete description of the model, specifying the exact formulation used in the present study. The firing rate variables r_k are indexed by $k = \{AB, A, B\}$ for each population shown in Fig 1A with the associated adaptation a_k and recurrent excitation e_k variables (note that the symbol "e" is used exclusively for excitation variables and associated constants whilst the symbol "exp ()" is used for the exponential function). The system of first order differential equations is as follows:

$$\begin{aligned}
\tau_r \dot{r}_{\mathrm{AB}} &= -r_{\mathrm{AB}} + F\Big(\beta_e e_{\mathrm{AB}} - \beta_i(r_{\mathrm{AB}} + r_{\mathrm{A}} + r_{\mathrm{B}}) - g a_{\mathrm{AB}} + I_{\mathrm{AB}} + \chi_{\mathrm{AB}}\Big),\\
\tau_r \dot{r}_{\mathrm{A}} &= -r_{\mathrm{A}} + F\Big(\beta_e e_{\mathrm{A}} - \beta_i(2r_{\mathrm{AB}} + r_{\mathrm{A}} + r_{\mathrm{B}}) - g a_{\mathrm{A}} + I_{\mathrm{A}} + \chi_{\mathrm{A}}\Big),\\
\tau_r \dot{r}_{\mathrm{B}} &= -r_{\mathrm{B}} + F\Big(\beta_e e_{\mathrm{B}} - \beta_i(2r_{\mathrm{AB}} + r_{\mathrm{A}} + r_{\mathrm{B}}) - g a_{\mathrm{B}} + I_{\mathrm{B}} + \chi_{\mathrm{B}}\Big),\\
\tau_a \dot{a}_{\mathrm{AB}} &= -a_{\mathrm{AB}} + r_{\mathrm{AB}},\\
\tau_a \dot{a}_{\mathrm{A}} &= -a_{\mathrm{A}} + r_{\mathrm{A}},\\
\tau_a \dot{a}_{\mathrm{B}} &= -a_{\mathrm{B}} + r_{\mathrm{B}},\\
\tau_{\mathrm{e}} \dot{e}_{\mathrm{AB}} &= -e_{\mathrm{AB}} + r_{\mathrm{AB}},\\
\tau_{\mathrm{e}} \dot{e}_{\mathrm{A}} &= -e_{\mathrm{A}} + r_{\mathrm{A}},\\
\tau_{\mathrm{e}} \dot{e}_{\mathrm{B}} &= -e_{\mathrm{B}} + r_{\mathrm{B}},
\end{aligned} \tag{1}$$

with time constants $\tau_r = 10\,\mathrm{ms}$ (cortical), $\tau_a = 1.4\,\mathrm{s}$ (spike frequency adaptation), $\tau_{\mathrm{e}} = 70\,\mathrm{ms}$ (NMDA-excitation). The strength of recurrent excitation is given by $\beta_e = 0.65$, lateral inhibition $\beta_i = 0.3$ and adaptation $g = 0.045$. Note that the profile of inhibition used here, with non-uniform synaptic weights and independent of DF, was determined after fitting the model to behavioural data (Rankin et al 2015). Note that although within-unit inhibition is included, $\beta_e > \beta_i$, so there is always net within-unit excitation. The firing rate function F is given by

$$F(u) = \frac{1}{1 + \exp\left(k_F(-u + \theta_F)\right)}, \tag{2}$$

where $\theta_F = 0.2$ is a threshold parameter and $k_F = 12$ is a slope parameter.

Experiment 1A: cond = {3 trip; 7 trip; 10 trip control}					
Effect	dfn	dfd	F	p [† if GG-corrected]	ges
DF	2	14	37.486	**0.000**	0.571
cond	2	14	19.486	**0.001**†	0.492
DF:cond	4	28	4.336	**0.007**	0.094
Experiment 1B: cond = {10 trip control; 300 ms gap; 600 ms gap}					
Effect	dfn	dfd	F	p [† if GG-corrected]	ges
DF	2	14	22.945	**0.001**†	0.603
cond	2	14	5.126	**0.021**	0.127
DF:cond	4	28	1.457	0.242	0.021
Experiment 2: cond = {6 trip control; disB+2; disB+15; devB+2}					
Effect	dfn	dfd	F	p [† if GG-corrected]	ges
DF	2	14	19.511	**0.000**	0.521
cond	3	21	5.796	**0.038**†	0.203
DF:cond	6	42	2.414	**0.043**	0.055
Experiment 3: cond = {6 trip control; disB+4; disB+8; disA-2 }					
Effect	dfn	dfd	F	p [† if GG-corrected]	ges
df	2	14	29.461	**0.000**†	0.624
cond	3	21	2.145	0.125	0.064
df:cond	6	42	0.873	0.523	0.017
Experiment 4: cond = {6 trip control; disB; disAB; disA}					
Effect	dfn	dfd	F	p [† if GG-corrected]	ges
DF	2	14	53.899	**0.000**	0.604
cond	3	21	5.002	**0.035**†	0.167
DF:cond	6	42	1.632	0.162	0.029
Experiments 2-4 compare controls: cond = {3 trip; 6 trip control}, exp = {2; 3; 4}					
Effect	dfn	dfd	F	p [† if GG-corrected]	ges
exp	2	18	0.057	0.945	0.003
DF	2	36	49.041	**0.001**†	0.491
cond	1	18	12.191	**0.003**	0.067
exp:DF	4	36	0.029	0.998	0.001
exp:cond	2	18	0.018	0.982	0.000
DF:cond	2	36	4.372	**0.020**	0.016
exp:DF:cond	4	36	0.906	0.471	0.007

Table 1: Analysis of Variance (ANOVA) tables for repeated measures experiments (N=8 subjects) shown in SIFig. 1. Columns are effect degrees of freedom (dfn), error degrees of freedom (dfd), F-value, p-value, generalized eta-squared effect size (ges). Significant p-values (0.05 significance level) are bold. A star indicates that the Greenhouse-Geiser corrected p-value was used due to Mauchly's sphericity test reaching significance at the $\alpha = 0.05$. In all experiments frequency difference conditions were DF ={4,7,10}. The first table (Experiment 1A) compares the control conditions of different lengths for Experiment 1 (SIFig. 1A). The second (Experiment 1B) compares the main 10 triplet control with the test conditions. Similarly for Experiments 2–4 comparing the 6 triplet control with the test conditions. The last table compares the 3 and 6 triplet control conditions across Experiments 2–4. Each experiment had a different set of N=8 subjects but we found no effect for exp (subject group), i.e. the subject groups gave similar results for the controls. This demonstrates that it is relevant to compare data from the test conditions in Experiments 2–4, as was done in Fig 4F of the main paper.

Additive noise is introduced with independent stochastic processes χ_{A}, χ_{B} and χ_{AB} added to the inputs of each population. Input noise is modeled as an Ornstien-Uhlenbeck process:

$$\dot{\chi_k} = -\frac{\chi_k}{\tau_d} + \gamma\sqrt{\frac{2}{\tau_\chi}}\xi_k(t), \tag{3}$$

where $\tau_\chi = 100\,\mathrm{ms}$ (a standard choice (Shpiro et al 2009; Seely and Chow 2011)) is the timescale, the strength γ equals 0.0875 and $\xi(t)$ is a white noise process with zero mean. Note these terms appear inside the firing rate function F such that firing rates r_k remain positive and do not exceed 1.

B.1 Model inputs and early adaptation

The particular form of the periodic inputs are based on recorded responses from A1 with ABA_ triplets (Micheyl et al 2005). We capture the basic form of these responses to tones (TR) with a pair of onset response functions, one with larger amplitude and early rise that captures the initial onset and a second with smaller amplitude and late rise that captures the plateau:

$$\mathrm{TR}(t) = H(t)\left[\frac{\exp(2)}{\alpha_1^2}t^2\exp\left(\frac{-2t}{\alpha_1}\right) + \Lambda_2\frac{\exp(2)}{\alpha_2^2}t^2\exp\left(\frac{-2t}{\alpha_2}\right)\right], \tag{4}$$

with plateau amplitude fraction $\Lambda_2 < 1$ and rise times $\alpha_1 < \alpha_2$. The constant terms $\frac{\exp(2)}{\alpha_{\{1,2\}}}$ terms normalise the amplitude at $t = \alpha_{\{1,2\}}$ of the individual onset functions to 1. A standard Heaviside function H (step function where $H(t) = 0$ zero for $t < 0$ and $H(t) = 1$ for $t > 0$) ensures no response before an input tone at $t = 0$. Rise times of $\alpha_1 = 15\,\mathrm{ms}$ and $\alpha_2 = 82.5\,\mathrm{ms}$ and an amplitude $\Lambda_2 = 1/6$ were chosen to approximately match the rise time and relative onset-to-plateau ratio observed in Micheyl et al (2005).

The spread of input is defined via the weighting function

$$w_p(\mathrm{DF}, t) = Q(t) I_p \exp\left(\frac{-R(t)\mathrm{DF}}{\sigma_p}\right), \tag{5}$$

where the tonotopic decay constant is $\sigma_p = 9.7\,\mathrm{st}$, the input amplitude is $I_p = 0.6$, $R(t)$ represents effective DF adaptation (increasing with time) and $Q(t)$ represents amplitude adaptation (decreasing with time). These are the two components of the early fast-adaptation in A1 sharing a common timescale $\tau_{\mathrm{A1}} = 500\,\mathrm{ms}$. The tonotopic spread of inputs in A1 evolves with time according to

$$R(t) = 1 - (1 - p)\exp(-t/\tau_{\mathrm{A1}}), \tag{6}$$

where the initial DF fraction is $p = 0.1$ ($R(t)$ rises from 0.1 to 1; effective DF rises from 0.1DF to DF). The input amplitude evolves according to

$$Q(t) = 1 + m\exp(-t/\tau_{\mathrm{A1}}), \tag{7}$$

where the $1 + m$ ($m = 2.5$) is the initial input amplitude factor ($Q(t)$ decays exponentially from 3.5 to 1; input amplitude decays from $3.5I_p$ to I_p).

In order to specify the amount of input received by each unit, I_{AB}, I_{A} and I_{B}, in (1), we first construct sequences of tone responses $\mathrm{TR}_A(t)$ (A_A_. . .) and $\mathrm{TR}_B(t)$ (_B__. . .) where the tones and silences ("_") each have a duration of 100 ms. Inputs for a repeating ABA_. . . sequence are given by

$$\begin{aligned} I_{\mathrm{AB}}(t) &= w(DF/2, t)(\mathrm{TR}_A(t) + \mathrm{TR}_B(t)),\\ I_{\mathrm{A}}(t) &= w(0, t)\mathrm{TR}_A(t) + w(DF, t)\mathrm{TR}_B(t),\\ I_{\mathrm{B}}(t) &= w(DF, t)\mathrm{TR}_A(t) + w(0, t)\mathrm{TR}_B(t), \end{aligned} \tag{8}$$

and plotted in Fig 1B. Respectively, equations (6) and (7) describe the early adaptation on the timescale τ_{A1} of the effective DF and the amplitude of responses in A1; see Fig 1B "Early adaption". After this initial adaptation during ~ 3 triplets, $w(DF, t_{\mathrm{late}}) = I_p\exp(-DF/\sigma_p)$ is independent of time; see Fig 1B "Static inputs". After a stimulus pause, both components recover on a timescale $\tau_{\mathrm{rec}} = 100\,\mathrm{ms}$. The amplitude component can recover completely (7) and the tonotopic spread partially recovers ($p = 0.325$ in (6), rather than 0.1); see Fig 3A.

We now specify how the formulation of the model in the present study relates to the one in Rankin et al (2015). In our previous study a slow synaptic depression on the recurrent excitation was introduced, but here we assume this does not play a role in the build up phase, i.e. we use static excitation (denoted e_{fix} in Rankin et al (2015)). To maintain a match to our experimental data under this assumption g, β_e, γ and I_p were adjusted relative to the values used in Rankin et al (2015). In the present study we use global, rather than DF-dependent, inhibition (denoted i_{gbl} in Rankin et al (2015)), see our previous paper for further discussion on this point. The input terms in (1) given by I_{AB}, I_{A}, I_{B} refer to the input to the competition stage, which may be different to the A1 responses, e.g. particular when inputs from distractor tones are gated out; see Fig 4A.

B.2 Inputs from distractor and deviant tones, simple implementation of SSA

For a distractor tone at tonotopic location d, or a deviant tone at tonotopic location D, the amplitude response in A1 can be computed in terms of the frequency difference $\mathrm{DF_d}$ (or $\mathrm{DF_D}$) between d (or D) and the tonotopic locations A, (A+B)/2 and B. The weighting function for a distractor (similarly for a deviant) is given by

$$w_{\mathrm{d}}(\mathrm{DF_d}, t) = I_{\mathrm{d}} Q(t)\exp\left(\frac{-\mathrm{DF_d}}{\sigma_{\mathrm{d}}}\right), \tag{9}$$

where, the amplitude adapts through $Q(t)$ and the tonotopic spread is assumed broad $\sigma_{\mathrm{d}} = 2.7\sigma_p$ (for example when above or below the A and B tones). In the presence of the ABA_ triplets, the A location is hit by more tones and, if a distractor immediately follows at A, it will be significantly adapted due to stimulus specific adaptation (SSA) Ulanovsky et al (2004); Taaseh et al (2011) in A1. As such, a relatively smaller response is assumed at the A location (factor 0.5 in (10)). This ad hoc, straightforward implementation of SSA is illustrated in SIFig. 2B. We provide a more general implementation of SSA below. We now let $\mathrm{TR_d}(t)$ (. . . ____ ___d ____. . .) represent an impulse (4) at the specific time of the additional distractor tone. A distractor tone, as a salient new event, is assumed boosted ($I_{\mathrm{d}} = 2.8I_p$) when it is it integrated as input to the

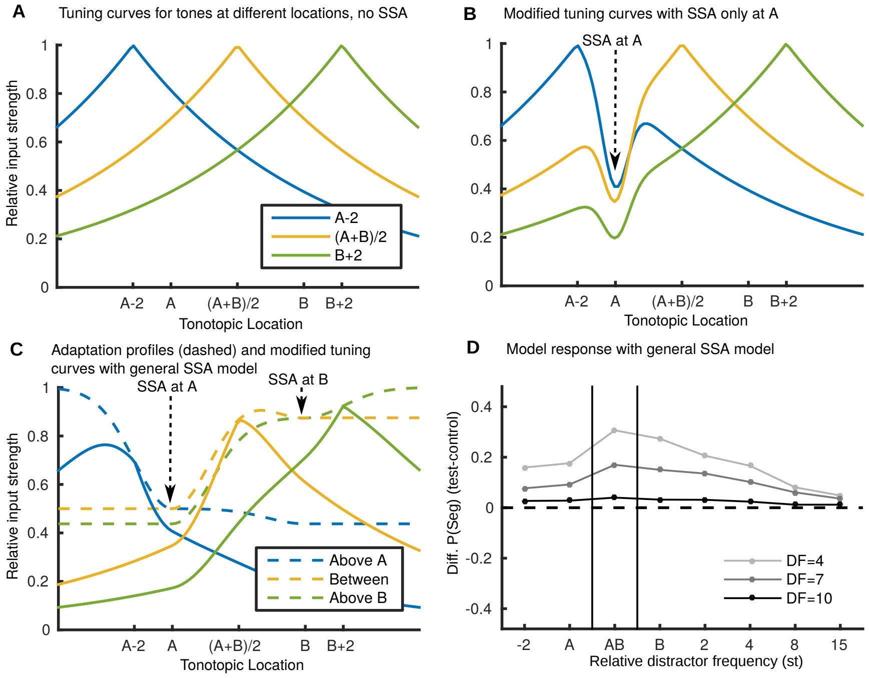

SIFig 2: Amplitude of A1 responses for distractor tones at different locations relative to A and B tones. (A) Tonotopic tuning of responses to tones in A1 at locations A-2, (A+B)/2 and B+2 without SSA (e.g. responses to isolated tones with no prior input). With no SSA, the tuned response is translated horizontally depending on the location of the tone. (B) Representative tuning curves with SSA only at the A location. (C) Tuning curves with general SSA model. More tones arrive at the A location and it will be more adapted than the B location. The profile of adaptation is shown for tones below (dashed blue), between (dashed yellow) or above (dashed green) the A and B tones. Solid curves show the tonotopic tuning of responses for tones at different location (legend in A); these are computed by multiplying the tuning curves in panel A with the adaptation profiles (dashed curves) in B. (D) As Fig 4E with general SSA model rather than SSA only at A location. Shows change in proportion segregated as a function of distractor location relative to A and B tones. Note x-axis does not have fixed spacing and distance between A and B changes with DF. Apart than the SSA model, the same assumptions are used (boost of inputs to A-unit and B-unit, no input to AB-unit (Fig 4A).

competition stage (see Fig 3D, where the distractor tone d gives larger amplitude input to the competition stage than preceding tones). For, say, a distractor tone at tonotopic location B+2 the modified inputs would be

$$\begin{aligned} \widehat{I_{\mathrm{AB}}}(t) &= I_{\mathrm{AB}}(t) + w_{\mathrm{d}}(\mathrm{DF}/2+2)\mathrm{TR}_{\mathrm{d}}(t), \\ \widehat{I_{\mathrm{A}}}(t) &= I_{\mathrm{A}}(t) + 0.5 w_{\mathrm{d}}(\mathrm{DF}+2)\mathrm{TR}_{\mathrm{d}}(t), \\ \widehat{I_{\mathrm{B}}}(t) &= I_{\mathrm{B}}(t) + w_{\mathrm{d}}(2)\mathrm{TR}_{\mathrm{d}}(t), \end{aligned} \tag{10}$$

see Fig 3D. For a deviant tone D we use the same rules ($w_{\mathrm{D}}(\mathrm{DF}_{\mathrm{D}}, t) = w_{\mathrm{d}}(\mathrm{DF}_{\mathrm{d}}, t)$), but the impulse $\mathrm{TR}_{\mathrm{D}}(t)$ (. . . ___ _D_ _ ___. . .) would replace a B tone in $\mathrm{TR}_{\mathrm{B}}(t)$. Incorporating the assumption illustrated in Fig 4A, that distractor tone responses in A1 do not propagate to the integrated unit, $\widehat{I_{\mathrm{AB}}}(t) = I_{\mathrm{AB}}(t)$ in (10); see Fig 4B.

B.3 General model for stimulus specific adaptation in A1

Here we provide a more general description of how neuronal responses in A1 depend on the tonotopic location of a new tone subject due to SSA from preceding tones. Our implementation of SSA is based primarily on feedforward effects. In SSA a location that has received a sustained input will be adapted in response to further input at the same tonotopic location, with a bandwidth of around 3–4 st in A1 (Ulanovsky et al 2004; Taaseh et al 2011). We provide a plausible, general implementation of SSA in our model, that could describe A1 responses and be used to determine the inputs from distractor tones to the model's competition stage. Then general schema described below for computing the relative amplitude of responses to new tones, additional to the ABA_ triplets yields very similar results to the ad hoc description above, compare Fig 4E with SIFig. 2D.

The general principal is to determine how the tuning curve for a new tone might be modified, based on previous inputs from the regular triplet tones. Example tuning curves for new tones (shown unadapted in SIFig. 2A), are modified by the

adaptation profiles (dashed curves in SIFig. 2B), dependent on the relative location of the new tone to preceding inputs. The adaptation profiles show the most adaptation close to the A tones (fast repetition rate), and less adaptation close to the B tones (slow repetition rate). For a new tone below A, the tuning curves (blue solid curve in SIFig. 2B) is carved out on the right hand side. For a new tone above B, the tuning curves (green solid curve in SIFig. 2B) is carved out on the left hand side. For a tone in between the tuning curve is carved out on either side (yellow solid curve in SIFig. 2B). Below we give a more complete, mathematical description of how the modified tuning curves are calculated.

In this more general formulation, functions will be defined in terms of a tonotopic coordinate y, rather than in terms of a frequency difference $\mathrm{DF_d}$, as used above in (9). In the absence of any prior input, an isolated tone will elicit a response in A1, largest at the tonotopic location of the tone, and decaying on either side (SIFig. 2A). In Rankin et al (2015), the tuning of these responses was assumed to have a symmetric exponential decay and, for a tone at a location N, this can be described by

$$TC(y,N) = \exp\left(\frac{-|y-N|}{\sigma_{\mathrm{tc}}}\right), \tag{11}$$

where $\sigma_{\mathrm{tc}} = 4\sigma_p$ is broad relative to the post-adaptation tuning width for the A and B tones in (5). In the presence of repeating ABA_ triplets that precede a new tone, the tuned responses will depend on the location of the new tone relative to the As or the Bs. In general, if a series of tones has been arriving at a specific tonotopic location L (either A or B) then the tuning curve of any subsequent tones will be altered. For a new tone N_+ above L the left side of its tuning curve will be reduced. For a new tone N_- below L the right side of its tuning curve will be reduced. The following equation describes the Gaussian *adaptation profile* AP around the L location

$$AP_+(y,L) = \begin{cases} 1 - c_L \exp\left(\frac{-(y-L)^2}{2(BW/2)^2}\right), & y < L \\ 1 - c_L, & y \geq L, \end{cases} \tag{12}$$

where $BW = 4$ is the bandwidth of adaptation and c_L is the amplitude of adaptation, which will be larger when, for example, the preceding sequence of L tones has a higher repetition rate. Equation 12 is 1 for $y \ll L$, decreases with Gaussian decay to $1 - c_L$ as y approaches L from below and is $1 - c_L$ for $y \geq L$. We similarly define AP for a tone below L

$$AP_-(y,L) = \begin{cases} 1 - c_L, & y \leq L \\ 1 - c_L \exp\left(\frac{-(y-L)^2}{2(BW/2)^2}\right) & y > L. \end{cases} \tag{13}$$

In this way the modified tuning curve $\widehat{TC}$ for a tone N_+ above L is given by multiplying the tuning curve with the appropriate adaptation profile

$$\widehat{TC}(y,N_+,L) = TC(y,N_+)AP_+(y,L), \tag{14}$$

and for a tone N_- below L is similarly given by

$$\widehat{TC}(y,N_-,L) = TC(y,N_-)AP_-(y,L). \tag{15}$$

If a tuning curve will be modulated by two sequences of tones L_1 and L_2, an additional argument in (14) or (15) can signify further modulation of the tuning curve by a second adaptation profile, e.g. $\widehat{TC}(y,N_-,L_1,L_2) = TC(y,N_-)AP_-(y,L_1)AP_-(y,L_2)$. These functions can now be used to work out the tuning curves for responses to deviant tones d, relative to the locations of the A and B tones featured in the ABA_ triplet sequence. Assuming significantly more adaptation at the A location due to the higher repetition rate, we set the adaptation strengths associated respectively with the A and B locations to be $c_A = 0.5$ and $c_B = 0.125$. The adaptation profile for a tone below A (which is also below B) will be

$$AP_{A-}(y,A,B) = AP_-(y,A)AP_-(y,B), \tag{16}$$

and is plotted dashed blue in SIFig. 2B. For a tone between A and B (above A and below B), we have

$$AP_{AB}(y,A,B) = AP_+(y,A)AP_-(y,B), \tag{17}$$

plotted dashed yellow in SIFig. 2B. For a tone above B (also above A), we have

$$AP_{B+}(y,A,B) = AP_+(y,B)AP_+(y,A), \tag{18}$$

plotted dashed green in SIFig. 2B. For example, the tuning curve for a new tone (e.g. distractor tone) arriving at a location A-2 (SIFig. 2B solid blue) is given by

$$\widehat{TC}_{A-}(y,A-2,A,B) = TC(y,A-2)AP_{A-}(y,A,B), \tag{19}$$

at a location (A+B)/2 (SIFig. 2B solid yellow) is given by

$$\widehat{TC}_{AB}(y,(A+B)/2,A,B) = TC(y,(A+B)/2)AP_{AB}(y,A,B), \tag{20}$$

and at a location B+2 (SIFig. 2B solid green) is given by

$$\widehat{TC}_{B+}(y,B+2,A,B) = TC(y,B+2)/2)AP_{B+}(y,A,B). \tag{21}$$

To summarise, for $\widehat{TC}$, the first argument is tonotopic location, the second argument the location of a new tone. The subscript $A-$, AB or $B+$ indicates whether the new tone is below, between, or above the A and B tones. The third and fourth arguments are the adapted locations for preceding tones (here A and B from the ABA_ triplets). Having defined the

relative amplitude across tonotopy in A1, we now describe the final steps to determine the inputs to the model's competition stage. Similar to (10), the inputs for, say, a distractor tone d above B

$$\begin{aligned}\widehat{I_{\text{AB}}}(t) &= I_{\text{AB}}(t) + I_{\text{ssa}}Q(t)\widehat{TC}_{B+}((A+B)/2, d, A, B),\\ \widehat{I_{\text{A}}(t)} &= I_{\text{A}}(t) + I_{\text{ssa}}Q(t)\widehat{TC}_{B+}(A, d, A, B),\\ \widehat{I_{\text{B}}(t)} &= I_{\text{B}}(t) + I_{\text{ssa}}Q(t)\widehat{TC}_{B+}(B, d, A, B),\end{aligned} \tag{22}$$

where $Q(t)$ describes early onset adaptation and $I_{\text{ssa}} = 3I_p$ is the boosted amplitude for a salient new tone. Again, if we were to incorporate the assumption illustrated in Fig 4A, that no input from a distractor tone reaches in AB-unit, we set $\widehat{I_{\text{AB}}(t)} = I_{\text{AB}}(t)$. SIFig. 2D shows the effect on proportion segregated of distractor tones at different tonotopic locations with the general model for SSA presented here. The general model for SSA captures the same features as show in Fig 4E, also based on the same assumptions illustrated in Fig 4A, but with a different implementation of SSA.

References

Howell DC (2013) Fundamental Statistics for the Behavioral Sciences. Cengage Learning

Lawrence MA (2013) ez: Easy analysis and visualization of factorial experiments. URL http://CRAN.R-project.org/package=ez, r package version 4.2-2

Micheyl C, Tian B, Carlyon R, Rauschecker J (2005) Perceptual organization of tone sequences in the auditory cortex of awake macaques. Neuron 48(1):139–148

R Core Team (2015) R: A Language and Environment for Statistical Computing. R Foundation for Statistical Computing, Vienna, Austria, URL https://www.R-project.org/

Rankin J, Sussman E, Rinzel J (2015) Neuromechanistic model of auditory bistability. PLOS Computational Biology 11(11):e1004,555

Seely J, Chow CC (2011) Role of mutual inhibition in binocular rivalry. Journal of Neurophysiology 106(5):2136–2150

Shpiro A, Moreno-Bote R, Rubin N, Rinzel J (2009) Balance between noise and adaptation in competition models of perceptual bistability. Journal of Computational Neuroscience 27:37–54

Taaseh N, Yaron A, Nelken I (2011) Stimulus-specific adaptation and deviance detection in the rat auditory cortex. PLOS ONE 6(8):e23,369–e23,369

Ulanovsky N, Las L, Farkas D, Nelken I (2004) Multiple time scales of adaptation in auditory cortex neurons. The Journal of Neuroscience 24(46):10,440–10,453
